# Highly sensitive multiplex PCR for convenient quantification and differentiation of canine *Oomycota* pathogens: *Pythium insidiosum*, *Lagenidium giganteum* f. *caninum,* and *Paralagenidium karlingii*

**DOI:** 10.1128/spectrum.03323-24

**Published:** 2025-04-24

**Authors:** Asfiha Tarannum, Subarna Barua, Leonel Mendoza, Raquel Vilela, Priscilla Barger, Terri Hathcock, Amelia White, Kelly Chenoweth, Chengming Wang

**Affiliations:** 1 Molecular Diagnostics Laboratory, College of Veterinary Medicine, Auburn University 70721, Auburn, Alabama, USA; 2 Department of Pathobiology, College of Veterinary Medicine, Auburn University 70721, Auburn, Alabama, USA; 3 Biomedical Laboratory Diagnostics, Michigan State University 3078, East Lansing, Michigan, USA; 4 Microbiology, Genetics and Immunology, Michigan State University 3078, East Lansing, Michigan, USA; 5 Pythium Serology & Mucormycosis Serology Laboratory, College of Veterinary Medicine, Auburn University 70721, Auburn, Alabama, USA; 6 Department of Clinical Sciences, College of Veterinary Medicine, Auburn University 70721, Auburn, Alabama, USA; Brown University, Providence, Rhode Island, USA

**Keywords:** *Pythium insidiosum*, *Lagenidium giganteum* f. *caninum*, *Paralagenidium karlingii*, PCR, *Oomycota*, dogs

## Abstract

*Oomycota* mammalian pathogens, including *Pythium* (*P*.) *insidiosum*, *Lagenidium* (*L*.) *giganteum* f. *caninum*, and *Paralagenidium* (*Para*.) *karlingii*, are increasingly recognized as causes of life-threatening infections in dogs, with diagnosis complicated by their clinical and phenotypic resemblance to fungal pathogens. Traditional diagnostic methods, such as serology, histopathology, and culture, often lack sensitivity and specificity, delaying appropriate management. Here, we developed a multiplex PCR assay with remarkable sensitivity, detecting as few as one DNA copy per reaction for *P. insidiosum* and *Para. karlingii* and 10 copies per reaction for *L. giganteum*. Validation on pure isolates from ATCC and USDA-ARS and other clinical isolates confirmed the assay’s specificity, correctly identifying all three pathogens and showing no cross-reactivity in non-target isolates. Interestingly, the developed multiplex *Oomycota* PCR revealed that 45.5% (40/88) of serum samples positive for anti-*Pythium* antibody contained *Pythium* DNA. Additionally, among 24 suspected anti-*Pythium* antibody-positive samples, 54.2% (13/24) tested positive for *Pythium* DNA, while 8.3% (2/24) contained *Lagenidium* DNA. By allowing simultaneous detection and differentiation of these pathogens in a single reaction, the multiplex PCR significantly reduces diagnostic time and resource requirements. This highly sensitive diagnostic tool holds promise for enhancing the speed and accuracy of *Oomycota* infection diagnosis in dogs, as well as other mammalian species, including humans. This could enable the prompt implementation of species-specific diagnosis followed by early intervention, ultimately resulting in improved clinical outcomes.

**IMPORTANCE:**

This study addresses a critical gap in the diagnosis of life-threatening infections caused by *Oomycota* pathogens*—Pythium insidiosum*, *Lagenidium giganteum* f. *caninum*, and *Paralagenidium karlingii*—in dogs. These pathogens, often misdiagnosed as fungal infections due to overlapping clinical and phenotypic features, require accurate differentiation for appropriate treatment. Current diagnostic methods, including serology, histopathology, and culture, are time-consuming, lack specificity, and are prone to inconclusive results. The newly developed multiplex PCR assay offers a transformative solution by enabling simultaneous detection and differentiation of these pathogens with remarkable sensitivity and specificity. This tool not only reduces diagnostic time but also enhances the accuracy of pathogen identification, paving the way for earlier intervention and improved clinical outcomes. Moreover, its potential application in other mammalian species, including humans, underscores its broader significance in managing *Oomycota* infections.

## OBSERVATION

*Oomycota* pathogens causing infections in dogs comprise *Pythium* (*P*.) *aphanidermatum*, *Pythium insidiosum*, *Pythium periculosum*, *Lagenidium giganteum* f. *caninum*, *Paralagenidium* (*Para*.) *karlingii,* and *Paralagenidium ajellopsis* (1–10), and these organisms represent a significant but often under-diagnosed cause of life-threatening infections in dogs. For years, *P. insidiosum* was considered the sole mammalian pathogenic oomycete affecting dogs, causing a disease known as pythiosis. However, the discovery of *Lagenidium* and *Paralagenidium* species as novel canine pathogens has broadened our understanding of this group of oomycetes (2, 3, 5, 7, 9). Furthermore, with the initial report of *Para. karlingii*, a pathogen morphologically and antigenically like *Lagenidium* spp., causing cutaneous to subcutaneous infections in dogs, and often leading to lesions with progressive and chronic development, complicates the separation of these three novel etiologies. Due to their similar phenotypic characteristics in the infected tissues (coenocytic hyphae and eosinophilic granulomas) and overlapping clinical presentations with true fungal infections, the early detection of these pathogens in the clinical laboratory is challenging (2, 4–6, 9–11).

Infections caused by these *Oomycota* pathogens in dogs are clinically significant, as these organisms cause severe and often fatal diseases (2, 4, 9–11). *P. insidiosum* in dogs primarily affects the skin and gastrointestinal tract, while *Lagenidium* species and *Paralagenidium* species predominantly cause progressive cutaneous and subcutaneous diseases (2, 10). Accurate and timely identification of the causative pathogen is crucial, as the clinical prognosis and therapeutic strategies vary significantly among these pathogens (2, 4, 6, 9, 11).

The current diagnostic approaches—serology, histopathology, and culture—are time-consuming, sometimes leading to inconclusive results, especially distinguishing between *P. insidiosum*, *Lagenidium* species, and *Paralagenidium* species. While serological tests such as ELISA can detect antibodies against *P. insidiosum*, this test is not available for these pathogens in most clinical laboratories, and cross-reactivity with true fungal pathogens could complicate the diagnosis (2, 4, 9, 11). Histopathological examination of the infected tissues can also be difficult, as the structures developed by the mammalian pathogenic oomycetes resemble the hyphal elements formed by filamentous fungal pathogens, especially those in the *Entomophthorales*, making differentiation challenging without molecular confirmation (2, 4, 9, 11). Additionally, culture methods for these fungal-like pathogens are slow and often require specific conditions for successful isolation and specialized personnel for their identification, further delaying diagnosis (4, 9, 11).

Molecular diagnostic methods, particularly PCR-based approaches, have shown promise for identifying these pathogens (12–27). However, current PCR assays lack the ability to simultaneously detect and differentiate between all three *Oomycota* pathogens, and most are limited to detecting only a single species or genus. Given the clinical importance of distinguishing among these pathogens for appropriate treatment, a more comprehensive and rapid diagnostic tool is needed.

This study aims to address these diagnostic gaps by developing a highly sensitive multiplex PCR assay capable of simultaneously detecting and differentiating *P. insidiosum*, *Lagenidium giganteum* f. *caninum*, and *Paralagenidium* species. The goal is to establish a rapid, reliable, and cost-effective diagnostic method that can be applied to tissue, blood, and serum specimens, thus improving the speed and accuracy of diagnosis. By offering a convenient and efficient method for differentiating these *Oomycota* pathogens, we hypothesized that this study would provide critical support for timely clinical decision-making and more effective treatment strategies for canine infections caused by these fungal-like pathogens.

*Pythium insidiosum* (*n* = 5), *L. giganteum* f. *caninum* and f. *giganteum* (*n* = 9), and *Paralagenidium* (*n* = 2) (Table 1) were kindly provided by Dr. Mendoza at Michigan State University and were used to validate the *Oomycota* multiplex PCR system in this work. These mammalian pathogen isolates were originally isolated from people, dogs, and cats, whereas the non-mammalian pathogens (*L. giganteum* f. *giganteum*) were acquired at the USDA-ARS Collection of Entomopathogenic Fungal Oomycetes Cultures (Table 1).

**TABLE 1:** Clinical isolates used to validate PCR in this study

Genus	Species	Source	Clinical ID; collection^*a*^
*Pythium*	*P. insidiosum*	Thailand human vascular pythiosis	MTPI 14^*b*^
	*P. insidiosum*	Australia horse case	MTPI 25 = ATCC 64221^*d*^
	*P. insidiosum*	South Carolina dog cutaneous lesions	MTPI 50
	*P. periculosum*	India human keratitis	MTPI 72
	*P. insidiosum*	Texan horse with subcutaneous pythiosis	MTPI 04
*Lagenidium*	*L. giganteum* f. *giganteum*	Mosquito larvae	ARSEF(USDA) 373 (type isolate, genome available: Lag_gig_ARSEF373_v1.0)^*e*^
	*L. giganteum* f. *giganteum*	Mosquito larvae	ARSEF(USDA) 749
	*L. giganteum* f. *giganteum*	Mosquito larvae	ARSEF(USDA) 2531
	*L. giganteum* f. *giganteum*	Mosquito larvae	ARSEF(USDA) 3327
	*L. giganteum* f. *giganteum*	Mosquito larvae	ATCC 36492
	*L. giganteum* f. *caninum*	Florida dog skin lesions	MTLA 01^*c*^
	*L. giganteum* f. c*aninum*	North Carolina dog skin lesions	MTLA 02
	*L. albertoi*	Human keratitis from Thailand	MTLA 13 (type isolate)
	*L. deciduum*	Virginia cat tail granuloma	MTLA 24
*Paralagenidum*	*Para. ajellopsis*	North Carolina dog subcutaneous infection	MTLA 26 (type isolate)
	*Para. karlingii*	Alabama dog with subcutaneous lesions	MTLA 28 = 1391 (genome available: ASM298042v1)

^*a*^ Michigan State University Collection of Mammalian Pathogenic Oomycetes (MTPI-MTLA), Dr. Mendoza’s Laboratory.

^*b*^ MTPI: MedTech *Pythium insidiosum* collection.

^*c*^ MTLA: MedTech *Lagenidium* and *Paralagenidium* species collection.

^*d*^ American Type Culture Collection.

^*e*^ USDA-ARS Collection of Entomopathogenic Fungal Cultures.

In addition, 5 ATCC isolates and 11 clinical isolates provided by the Bacteriology and Mycology Diagnostic Laboratory at the Auburn University College of Veterinary Medicine were included in this work to validate the assay: *Aspergillus fumigatus, Aspergillus flavus* ATCC 16883*, Aspergillus terreus, Aspergillus niger* ATCC 16888*, Bipolaris* sp.*, Candida albicans, Candida parapsilosis* ATCC 22019*, Conidiobolus* sp.*, Cryptococcus laurentii* ATCC 18803*, Curvularia* sp.*, Fusarium* sp.*, Microsporum canis, Nocardia asteroids, Paeciliomyces variotii* ATCC 26820*, Paeciliomyces, Papiliotrema laurentii* ATCC 18803*, Prototheca* sp.*,* and *Sporothrix schenckii*.

Among the serum samples submitted to the *Pythium* Serology & Mucormycosis Serology Laboratory at the Auburn University College of Veterinary Medicine in 2023–2024, 88 randomly selected anti-*Pythium* antibody positive samples and 24 suspected anti-*Pythium* antibody-positive samples were used in this study.

Fungi from the pure culture and canine sera were homogenized in a shaker (Bertin Technologies, France) with four 3.0 mm ceramic beads for two periods of 15 s (3,160 *g* with a 15 s break in between). Total nucleic acid extraction was performed with glass fiber matrix binding and elution with a commercial kit (High-Pure PCR Template Preparation Kit; Roche Diagnostic) following the manufacturer’s instructions and described previously (28).

All oligonucleotides used in this study were designed by using the Vector NTI software (Invitrogen Corporation, Carlsbad, CA, USA). All available nucleotide sequences for the 18S rRNA of *P. insidiosum*, *L. giganteum*, *Para. karlingii,* and other fungi (*Aspergillus, Blastomyces, Candida, Cladophialophora, Coccidioides, Cryptococcus, Geotrichum, Histoplasma, Madurella, Malassezia, Microsporum, Nannizzia, Rhinosporidium, Sporothrix,* and *Trichophyton*) were obtained from GenBank. The nucleotide sequences for all these *Oomycota* pathogens and fungi were aligned for the identification of maximally conserved regions of the individual *Oomycota* pathogen, but significantly different from those of other *Oomycota* pathogens and fungi. This approach clearly identified the ITS1-5.8S-ITS2 region as the best target for designing three PCRs, specifically amplifying *P. insidiosum*, *L. giganteum,* or *Para. karlingii*. Comparison of all primers and probes for three *Oomycota* pathogens with all available 18S rRNA sequences confirmed that each set of primers and probe has 100% nucleotide similarity with the sequences of the designated *Oomycota* organism but demonstrated more than 60% dissimilarities in nucleotide sequences with two other *Oomycota* pathogens and other fungi. The nucleotide sequences of primers and probes are listed in Table 2. The *Oomycota* qPCR was performed in a LightCycler 96 PCR instrument (Roche) using a thermal protocol and PCR conditions optimized (28) with an annealing temperature of 57°C.

**TABLE 2:** Primers and probes used in this study

Oomycota	Primers and probes (5′−3′)	Region and amplicon size
*Pythium*	Upstream primer GTTGCAGCTGACGGGGTGTTGTTT	ITS2, 151 bp
	Downstream primer GTTCCCAAATTAGTGTCGTCCTCTTT	
	Probe /56-FAM/GT GTG TGA G/ZEN/G TCG AAC TGG ACG CT/3IABKFQ/	
*Lagenidium*	Upstream primer CTTGTTTTGTGCGCGAATGC	ITS1; 81 bp
	Downstream primer AAGTTCATCAGTCAATGCCAGCTT	
	Probe /5HEX/AC GYG CTG A/ZEN/A CGA AGG TTA GTG TTG /3IABkFQ/	
*Paralagenidium*	Upstream primer TTTGGAGAGCGCGTGCGA	ITS2; 108 bp
	Downstream primer CGAGGCAAACATGCGCTTC	
	Probe /5Cy5/TT TGC GGC G/TAO/AGTC CTT TTA AAT GCA /3IAbRQSp	

The gBlock gene fragments containing the whole length of the ITS1-5.8S-ITS2 region of *P. insidiosum*, *L. giganteum,* and *Para. karlingii* were synthesized by Integrated DNA Technologies (Coralville, IA, USA). Based on the molecularity of each gene fragment, dilutions were made to give solutions containing 10^4^, 10^3^, 10^2^, 10^1^, and 10^0^ gene copies per reaction. These were amplified with *Oomycota* qPCR in triplicates to determine the detection limit of the PCRs.

The specificity of the *Oomycota* qPCR was verified using the gene fragments with the whole length of the ITS1-5.8S-ITS2 region of *P. insidiosum*, *L. giganteum,* and *Para. karlingii*. To further confirm the specificity, the multiplex PCR was performed on the DNAs from the 16 pure isolates (Table 1) and an additional 16 fungal isolates other than these three *Oomycota* pathogens. The PCR products were sent to ELIM Biopharmaceuticals (Hayward, CA, USA) for Bidirectional Sanger sequencing.

**Fig 1:**
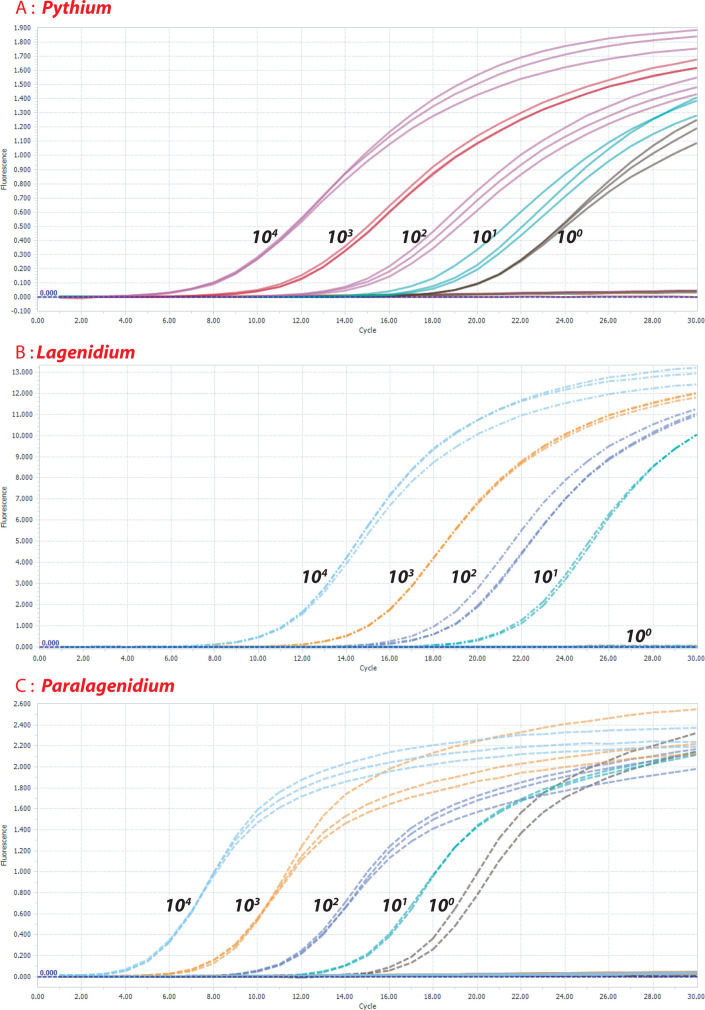
Highly sensitive and specific detection of *P. insidiosum*, *L. giganteum,* and *Para. karlingii* by a multiplex PCR. The established multiplex PCR in this study was used to amplify all quantitative standards (10^4^, 10^3^, 10^2^, 10^1^, and 10^0^ copies of the rRNA targets of *P. insidiosum*, *L. giganteum,* and *P. karlingii*). The PCR was found to amplify only the specific target, not two others. The detection limit was found to be one copy/reaction for *P. insidiosum* (**A**), *P. karlingii* (**C**), and 10 copies/reaction for *L. giganteum* (**B**).

The multiplex PCR established in this study showed sensitivity up to one copy per reaction for *P. insidiosum* and *Para. karlingii* and 10 copies per reaction for *L. giganteum* (Fig. 1). DNA sequencing confirmed that multiplex PCR identified the genus level of *Pythium*, *Lagenidium*, and *Paralagenidium* in 16 pure isolates in this study. None of these 16 isolates, other than these three *Oomycota* pathogens, were found to be PCR positive in this study. The multiplex PCR identified specifically the designated target. PCRs on the whole panels of the quantitative standards for all three *Oomycota* pathogens did not demonstrate cross talk between different fluorescent channels.

Interestingly, the multiplex *Oomycota* PCR developed in this study revealed that 45.5% (40/88) of serum samples positive for anti-*Pythium* antibody contained *Pythium* DNA. Additionally, among 24 suspected anti-*Pythium* antibody-positive samples, 54.2% (13/24) tested positive for *Pythium* DNA, while 8.3% (2/24) also proved to contain *Lagenidium* DNA.

The multiplex PCR method developed in this study offers a significant advancement in the rapid and accurate detection of three major Oomycota pathogens—*Pythium insidiosum*, *Lagenidium giganteum*, and *Paralagenidium*—infecting dogs. This method combines high sensitivity, excellent specificity, and the simultaneous detection of multiple pathogens in a single reaction, offering clear advantages over existing diagnostic approaches, such as serology, histopathology, and culture.

One of the primary strengths of this multiplex PCR is its high sensitivity, capable of detecting as few as one copy of DNA per reaction for *P. insidiosum* and *P. karlingii*, and 10 copies per reaction for *L. giganteum*. This sensitivity is particularly valuable in clinical settings, where early detection of these pathogens is critical for initiating timely treatment. In contrast, traditional methods such as serology are often less sensitive (2, 4, 9, 11), with antibody titers taking time to develop after infection and sometimes providing inconclusive results due to cross-reactivity with other fungal pathogens. Diagnoses and treatments may be delayed by techniques like culture-based identification and ITS sequencing, which are time-consuming and require significant expertise (29).

The low sensitivity of current diagnostic methods for detecting *Oomycota* pathogens in canine clinical samples is influenced by several factors. These include challenges in simultaneously detecting pathogens, limitations in diagnostic technologies, and variability in pathogen presence and distribution within samples (30). All these factors collectively hinder the accurate and timely diagnosis of *Oomycota* infections in dogs. Addressing this issue, the multiplex PCR in this study offers a significant improvement, with its specificity confirmed by the ability to detect only target pathogens, as evidenced by the absence of cross-reactivity with 16 non-target isolates tested. This superior level of specificity, combined with high sensitivity, makes PCR positivity a more reliable diagnostic indicator of infection compared to serology, which is prone to false positives or fails to detect early-stage infections.

Another significant advantage of this method is the simultaneous detection of three *Oomycota* pathogens in a single reaction, which saves both time and resources compared to running separate PCR assays for each pathogen. This multiplex approach not only streamlines the diagnostic process but also reduces the likelihood of missing a pathogen in cases of co-infection, where multiple *Oomycota* pathogens may be present. The ability to distinguish between *P. insidiosum*, *L. giganteum*, and *Para. karlingii* based on their unique fluorescence channels further enhances the utility of this method for clinical diagnosis.

Historically, one of the challenges with multiplex PCR systems has been fluorescence spillover, where there is unwanted “cross talk” between the detection channels, leading to inaccurate results (20, 26). This issue often necessitates extensive color compensation during data analysis. However, with recent advancements in PCR instrumentation, such as the PCR machine (Roche LightCycler 96 PCR instrument) used in this study, no spillover was observed. The ability to detect three targets without interference between channels is a significant technological improvement, ensuring more accurate and reliable results with minimal post-PCR adjustments.

Despite the promising results obtained in this study, there are some limitations that should be acknowledged. Clinical validation on actual patient samples remains an important next step. Although this study successfully tested the multiplex PCR on 16 pure isolates of the target pathogens and 16 pure isolates of other fungi, the method has yet to be tested on clinical samples from naturally infected dogs. Ideally, future studies should include testing clinical specimens from dogs that have been experimentally infected with *P. insidiosum*, *L. giganteum*, and *Para. karlingii*, as well as samples from naturally infected animals, to further confirm the assay’s diagnostic accuracy and reliability in real-world settings.

While this multiplex PCR assay represents a valuable tool for differentiating *P. insidiosum*, *L. giganteum*, and *P. karlingii*, targeting the highly conserved 18S rRNA gene, its specificity may be limited due to the potential amplification of the target gene in related species within these genera. For example, this assay, originally designed to detect *P. insidiosum*, was also found to successfully amplify *P. periculosum*, an important human pathogen. Therefore, while this assay provides genus-level identification for these *Oomycota* pathogens, it may not be sufficient to confirm species identity in all cases. For a more comprehensive species-level identification, additional techniques such as full sequencing of the rRNA gene and cytochrome c oxidase subunit I gene, whole-genome sequencing, or analysis of alternative genetic markers are recommended, especially in cases where morphological features are ambiguous (31, 32). Combining these approaches with morphological analysis can enhance diagnostic accuracy, ensuring more precise identification and better informing treatment strategies for infections caused by other potential *Oomycota* species within these genera (2).

An intriguing finding of this study was the detection of *Pythium* and *Lagenidium* DNA in anti-*Pythium* antibody-positive and suspect-positive sera, a result that is both unexpected and notable. *Pythium* infections are typically classified into two forms: cutaneous and gastrointestinal. Detection of *Pythium* DNA in sera is uncommon and suggests that the multiplex *Oomycota* PCR developed in this study possesses exceptional sensitivity. This heightened sensitivity could allow for the identification of circulating *Pythium* DNA that might otherwise remain undetected. Given the preliminary nature of this work, further studies with a larger sample size are planned to explore the relationship between PCR positivity (including DNA copy number) and ELISA results (including antibody titer), as well as to investigate potential correlations with infection stage and type (cutaneous or gastrointestinal pythiosis) and treatment status in affected dogs. These future efforts aim to enhance our understanding of the diagnostic utility of this PCR and its clinical implications.

In conclusion, the multiplex PCR assay developed in this study provides a highly sensitive, specific, and efficient diagnostic tool for detecting and differentiating *P. insidiosum*, *L. giganteum*, and *Para. karlingii* infections in dogs. This method outperforms traditional diagnostic techniques by offering faster results, higher sensitivity, and greater specificity, with the added benefit of simultaneous detection of multiple pathogens in a single reaction (13–16, 20–23, 25–27). The ability to detect these pathogens with high accuracy is crucial for timely intervention and appropriate treatment. Given its advantages, this multiplex PCR holds the potential to become an essential tool in veterinary diagnostics, enabling more rapid and reliable identification of *Oomycota* infections and ultimately improving clinical outcomes for affected dogs. Future studies are required to further validate this method in clinical settings, but the promising results presented here suggest that it will significantly enhance the diagnosis and management of *Oomycota* infections in veterinary practice.
